# THE AGING PROFESSOR OF GERONTOLOGY: MINING THE PLATINUM

**DOI:** 10.1093/geroni/igad104.1102

**Published:** 2023-12-21

**Authors:** Laura Donorfio, Lyn Holley, Becca Levy

## Abstract

This symposium explores evidence of the subtle, pernicious influence of unconsciously internalized ageist stereotypes on the engagement, job performance, perception of self and life satisfaction of older persons who are teachers of Gerontology in higher education. Unsolicited responses to the Tibbitts Award Lecture at the 2022 Conference of the Gerontological Society of American inspired this symposium and comments that indicated many older teachers of Gerontology in institutions of higher learning had become more aware of how internalized ageism and the ageism of others influences one’s pedagogy of aging. This includes one’s gerontological imagination, self-disclosure, thoughts about retirement, and organizational self-esteem. Learning to accept one’s own aging, personal growth, and activities to maintain professional competence are challenges to continuing in one’s role. This symposium captures the reflections of four established Gerontologists about these issues and how they shape careers, especially late life work. Results of a scoping literature review and some case studies of topics relevant to breaking the age code are reported. Topics to be covered include: the intersection of personal aging and aging pedagogy, career self-management of late life work, assessing and dealing with career competence and incompetence in late life work, and the Role of Age Friendly Environments. The discussant will capture the impact of ageism on these topics, and how working on beliefs about aging can influence and improve late life work and empower a productive lifespan.

